# Self-Assembly and Anti-Amyloid Cytotoxicity Activity of Amyloid beta Peptide Derivatives

**DOI:** 10.1038/srep43637

**Published:** 2017-03-08

**Authors:** V. Castelletto, P. Ryumin, R. Cramer, I. W. Hamley, M. Taylor, D. Allsop, M. Reza, J. Ruokolainen, T. Arnold, D. Hermida-Merino, C. I. Garcia, M. C. Leal, E. Castaño

**Affiliations:** 1 School of Chemistry, Pharmacy and Food Biosciences, University of Reading, Whiteknights, Reading RG6 6AD, UK; 2 Division of Biomedical and Life Sciences, Faculty of Health and Medicine, Lancaster University, Lancaster LA1 4YQ, UK; 3 Department of Applied Physics, Aalto University School of Science, Aalto FI-00076, Finland; 4 Diamond Light Source Ltd., Harwell Science and Innovation Campus, Didcot OX11 0DE, UK; 5 European Synchrotron Radiation Facility, ESRF, 71 avenue des Martyrs, 38000 Grenoble, France; 6 Fundación Instituto Leloir and Instituto de Investigaciones Bioquímicas de Buenos Aires, Consejo Nacional de Investigaciones Científicas y Técnicas, Buenos Aires, Argentina

## Abstract

The self-assembly of two derivatives of KLVFF, a fragment Aβ(16–20) of the amyloid beta (Aβ) peptide, is investigated and recovery of viability of neuroblastoma cells exposed to Aβ (1–42) is observed at sub-stoichiometric peptide concentrations. Fluorescence assays show that NH_2_-KLVFF-CONH_2_ undergoes hydrophobic collapse and amyloid formation at the same critical aggregation concentration (*cac*). In contrast, NH_2_-K(Boc)LVFF-CONH_2_ undergoes hydrophobic collapse at a low concentration, followed by amyloid formation at a higher *cac*. These findings are supported by the β-sheet features observed by FTIR. Electrospray ionization mass spectrometry indicates that NH_2_-K(Boc)LVFF-CONH_2_ forms a significant population of oligomeric species above the *cac*. Cryo-TEM, used together with SAXS to determine fibril dimensions, shows that the length and degree of twisting of peptide fibrils seem to be influenced by the net peptide charge. Grazing incidence X-ray scattering from thin peptide films shows features of β-sheet ordering for both peptides, along with evidence for lamellar ordering of NH_2_-KLVFF-CONH_2_. This work provides a comprehensive picture of the aggregation properties of these two KLVFF derivatives and shows their utility, in unaggregated form, in restoring the viability of neuroblastoma cells against Aβ-induced toxicity.

The Amyloid β (Aβ) peptide plays a key role in Alzheimer’s disease (AD), which is an increasingly prevalent condition in the aging population and as such is a major global healthcare challenge. There is thus intense research activity on the role of the Aβ peptide in causing neuronal cell death and in the progression of AD and its eventual treatment^1^,^2^,^3^,^4^,^5^,^6^,^7^,^8^,^9^,^10^. It is believed that aggregates of Aβ peptide, misfolded into β-sheet fibrils (so-called “amyloid”) are implicated in this process since clumps of aggregates termed plaques have been observed in brain tissue from AD sufferers. Oligomeric aggregates are believed to have higher cytotoxicity than fibrillar aggregates^11^,^12^,^13^,^14^,^15^. One strategy to potentially treat AD is to hinder or disrupt aggregation, or more specifically to disrupt the formation of oligomers.

A key sequence driving aggregation in Aβ is the core sequence Aβ^16^,^17^,^18^,^19^,^20^, KLVFF, which contains the diphenylalanine sequence which plays a significant effect in its aggregation propensity^10^. The aggregation of KLVFF has been investigated previously^16^,^17^,^18^,^19^,^20^,^21^,^22^,^23^,^24^,^25^,^26^,^27^,^28^ and many variants have been prepared in order to examine their influence on the aggregation of Aβ itself^10^,^29^. In a previous paper we examined the self-assembly of the KLVFF peptide with uncapped termini, NH_2_-KLVFF-COOH^30^, and showed for the first time clear evidence (in particular using cryogenic-TEM and small-angle X-ray scattering) that this molecule itself forms highly extended fibrils, with β-sheet structure (confirmed via FTIR spectroscopy and X-ray diffraction). The CD spectrum is dominated by the contribution of the π-stacking phenylalanine units which masks the typical spectrum of a β-sheet structure^10^,^29^,^30^. Some of us have previously reported a peptide inhibitor, (Ac-rGFFVLKGr-NH2, r denotes d-arginine) consisting of the retro-inverted version of KLVFF flanked by the solubilizing residues rG and Gr, that blocks the formation of Aβ oligomers and fibrils *in vitro* and also inhibits the toxic effects of Aβ on cell cultures^31^.

Here, we examine the self-assembly of the KLVFF peptide with C-terminal amidation, NH_2_-KLVFF-CONH_2_ (peptide **1**) compared to the lysine capped analogue NH_2_-K(Boc)LVFF-CONH_2_ (peptide **2**). The two peptides are shown in Fig. 1. In the latter peptide the lysine residue is capped with a Boc (*tert*-butyloxylcarbonyl) unit. In order to examine the influence of the lysine group and electrostatics on aggregation behaviour and bioactivity, we compare the self-assembly and cytotoxicity of peptide **1** with that of the homologue peptide **2**. A range of spectroscopy, microscopy and scattering techniques are used to investigate peptide self-assembly. Electrospray ionisation mass spectrometry is used to probe oligomeric species. Neurotoxicity assays are performed on Aβ (1–42)/peptide mixtures in order to assess the utility of these samples as therapeutic agents.

## Results

### Critical Aggregation Concentration

**Figure 2:**
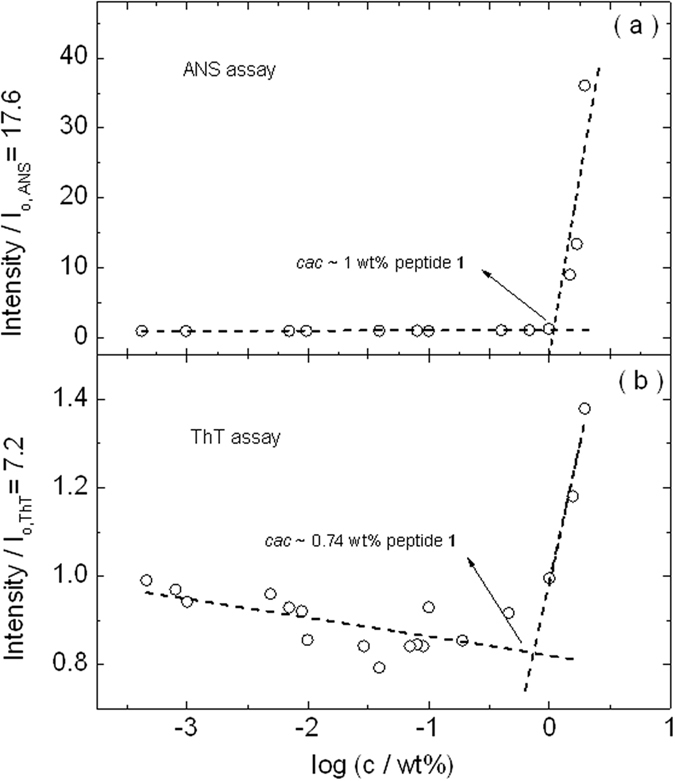
Critical aggregation concentration (*cac*) assay for peptide **1** using concentration-dependent (**a**) ANS and (**b**) ThT fluorescence assays.

Two types of fluorescence assays were used to determine critical aggregation concentration (*cac*) values. The first uses ANS, the fluorescence of which depends on the hydrophobic environment^32^,^33^,^34^,^35^,^36^. The second uses ThT, which is sensitive to the formation of amyloid fibrils^32^,^37^,^38^,^39^,^40^. Results of the two fluorescence assays for peptide **1** are shown in Fig. 2. The *cac* value obtained from the discontinuity in fluorescence intensity using ThT is in agreement within uncertainty with that from the ANS assay (1.00 ± 5 × 10^−2^) wt%. This indicates that for this peptide, hydrophobic collapse occurs concurrently with the formation of amyloid fibrils.

**Figure 3:**
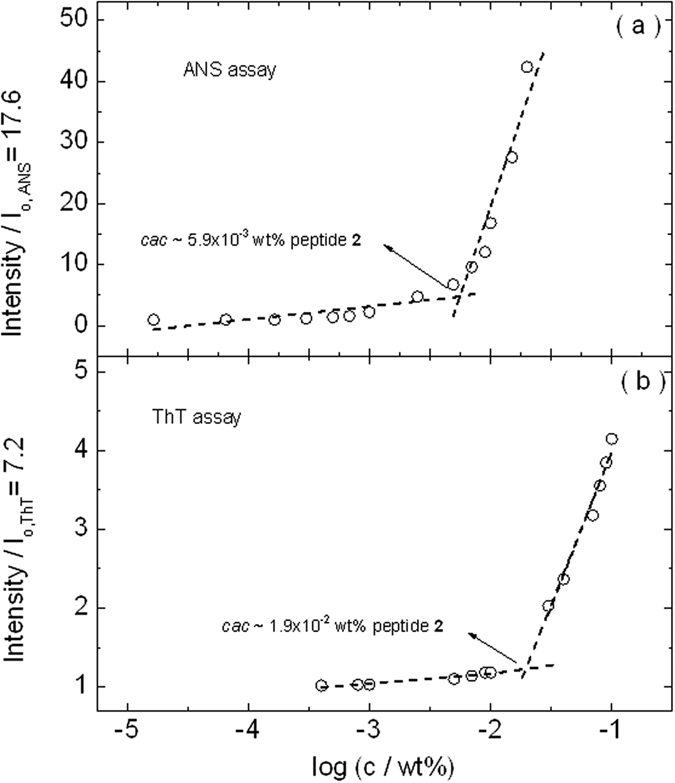
Critical aggregation concentration (*cac*) assay for peptide **2** using concentration-dependent (**a**) ANS and (**b**) ThT fluorescence assays.

The concentration-dependent fluorescence results for peptide **2** are shown in Fig. 3. The data shows firstly that the *cac* is substantially lower than for peptide **1**, due to the presence of the additional hydrophobic Boc group. In addition, significantly different values are obtained for the *cac* from the two fluorescence assays. This suggests that hydrophobic collapse occurs before formation of amyloid fibrils for sample **2** and that in between the two *cac* values (5.9 ± 0.1) × 10^−3^ wt% and (1.9 ± 0.1) × 10^−2^ wt% oligomers may be present.

### Electrospray Ionization Mass Spectrometry

ESI experiments were performed at 0.029 wt% to investigate the peptide’s aggregation stability. According to the results above, 0.029 wt% is below the *cac* for **1** but at the onset of peptide aggregation for **2**. ESI studies were first performed for peptides dissolved in water. Subsequently, the same study was repeated for peptides dissolved in HFIP: water (1:9; v/v). HFIP was added to test the stability of the peptide aggregates, since this organic solvent is routinely used to disrupt Aβ oligomerization in solution^41^.

**Figure 4:**
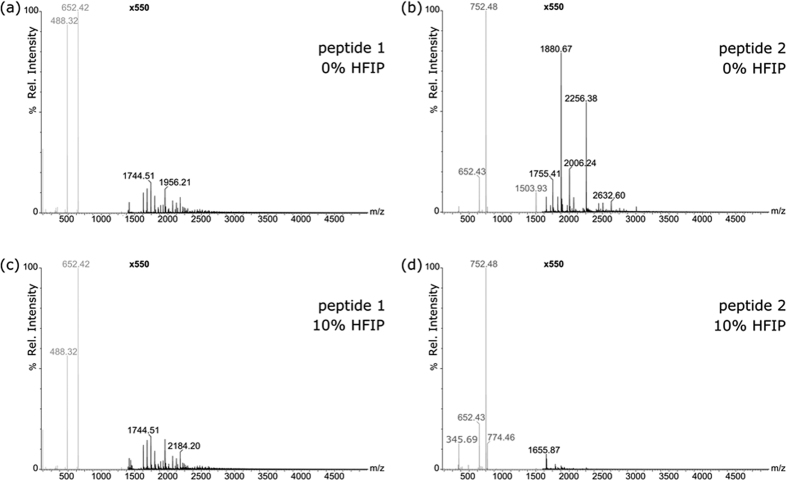
ESI mass spectra showing peptide aggregation stability for peptide **2** and peptide **1** with and without HFIP. (**a**) ESI mass spectrum of peptide **1** diluted in H_2_O to 2.9 × 10^−2^ wt%. (**b**) ESI mass spectrum of peptide **2** diluted in H_2_O to 2.9 × 10^−2^ wt%. (**c**) ESI mass spectrum of peptide **1** diluted in 10% HFIP to 2.9 × 10^−2^ wt%. (**d**) ESI mass spectrum of peptide **2** diluted in 10% HFIP to 2.9 × 10^−2^ wt%. The spectrum section shown in black is magnified 550 times.

ESI mass spectra showing peptide aggregation stability for **1** and **2** with and without HFIP are displayed in Fig. 4. Figures S1 and S2 show the ESI mass spectra with further peak annotation for peptide aggregation for **1** and **2**, respectively.

Figures 4a,c and S1 show that **1** at a concentration of 0.029 wt% forms only a few self-assemblies of low aggregation state (up to n = 7). The relative extents of oligomerisation of **1** measured in experiments without and with HFIP are (*Eo*)_*H*2*O*_ = 0.040 ± 0.002 and (*Eo*)_*HFIP*_ = 0.046 ± 0.002, respectively. This data shows that the oligomers at such low concentrations are stable upon HFIP addition. Figures 4b,d show that at 0.029 wt% **2** forms more oligomers than **1** at a much higher aggregation state (up to n = 20) which are not stable upon HFIP addition. The corresponding relative extents of oligomerisation of **2** measured in experiments without and with HFIP are (*Eo*)_*H*2*O*_ = 0.25 ± 0.002 and (*Eo*)_*HFIP*_ = 0.027 ± 0.004, respectively. As a whole, the ESI results are consistent with the *cac* assays in Figs 2 and 3. They show that there is little peptide aggregation for **1** below the *cac*, while for **2** extensive oligomerisation starts at this concentration.

### Secondary Structure and Aggregation

**Figure 5:**
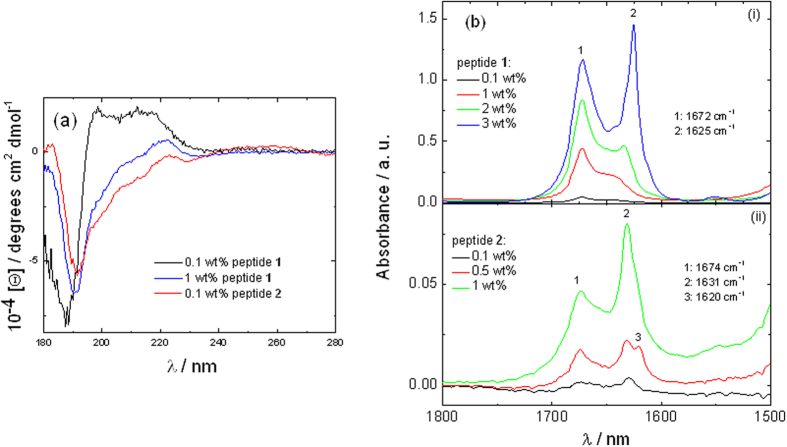
(**a**) CD spectra for sample **1** and sample **2** peptide solutions. (**b**) FTIR data measured for (i) sample **1** and (ii) sample **2** peptide solutions.

The secondary structure formed below and above the *cac* of sample **1** and above both *cacs* of sample **2** was investigated using circular dichroism (CD) and FTIR spectroscopies. CD spectra are shown in Fig. 5a. They do not show the shape associated with any canonical protein/peptide secondary structure. Instead, each spectrum is dominated by a minimum near 190 nm and a positive maximum near 220 nm. For peptide **1** it was also possible to measure a spectrum below the *cac* which differs from that obtained above the *cac* as it contains an additional maximum near 200 nm. The 220 nm maximum in the spectra (along with the 190 nm minimum) is characteristic of π-stacked phenylalanine residues^30^,^42^,^43^. This points to the presence of such interactions in whatever underlying secondary structure is present. This was probed using FTIR spectroscopy and fibre X-ray diffraction.

FTIR spectra are shown in Fig. 5b. For peptide **1**, the development of the peak at 1625 cm^−1^ for concentrations of 1 wt% and above (i.e. above the *cac*) is consistent with the development of β-sheet structure^44^,^45^,^46^. All the FTIR spectra exhibit a sharp peak at 1672 cm^−1^ due to bound TFA counterions^47^,^48^,^49^. For sample **2**, peaks in the 1620–1630 cm^−1^ range exist even from the lowest concentration for which FTIR spectra could be obtained (0.1 wt%) consistent with the relatively low *cac* value (even upper *cac* value) for this sample. This shows the presence of β-sheet structures for this peptide.

**Figure 6:**
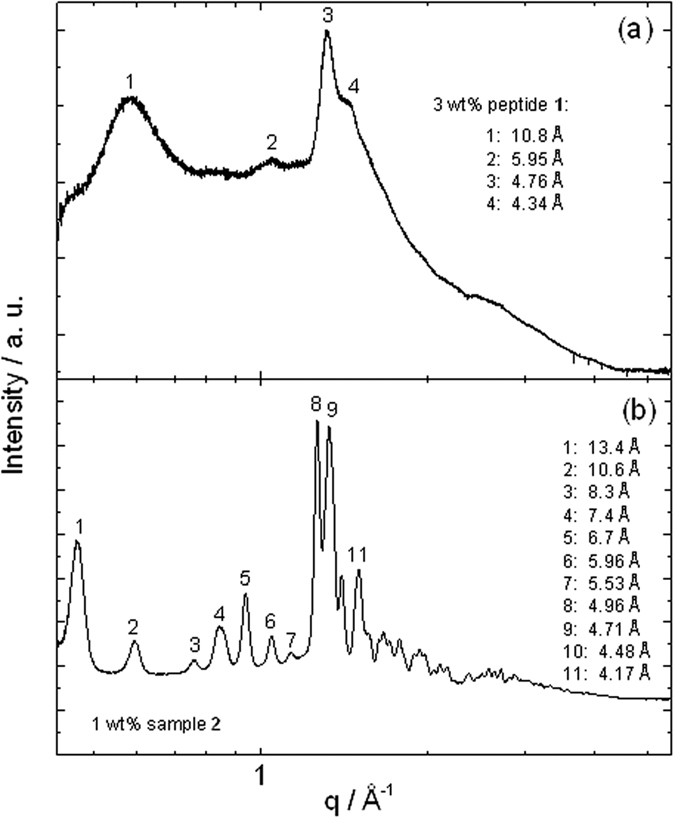
XRD profiles from dried stalks prepared from (**a**) 3 wt% sample **1** and (**b**) 1 wt% sample **2**, with principal peaks labelled.

Fibre X-ray diffraction intensity profiles are shown in Fig. 6. For sample **1**, the peaks can be associated with a typical cross-β XRD pattern^50^,^51^ due to the presence of reflections at 10.8 Å and 4.76 Å which are due respectively to the spacing of β-sheets and the separation of β-strands within the β-sheets. However, for sample **2**, cross-β pattern peaks at 10.6 Å and 4.71 Å coexist with a series of sharp peaks which are due to stacking of the Boc units. A very similar series of peaks was reported by us previously for Fmoc-K (Boc) LV^43^.

**Figure 7:**
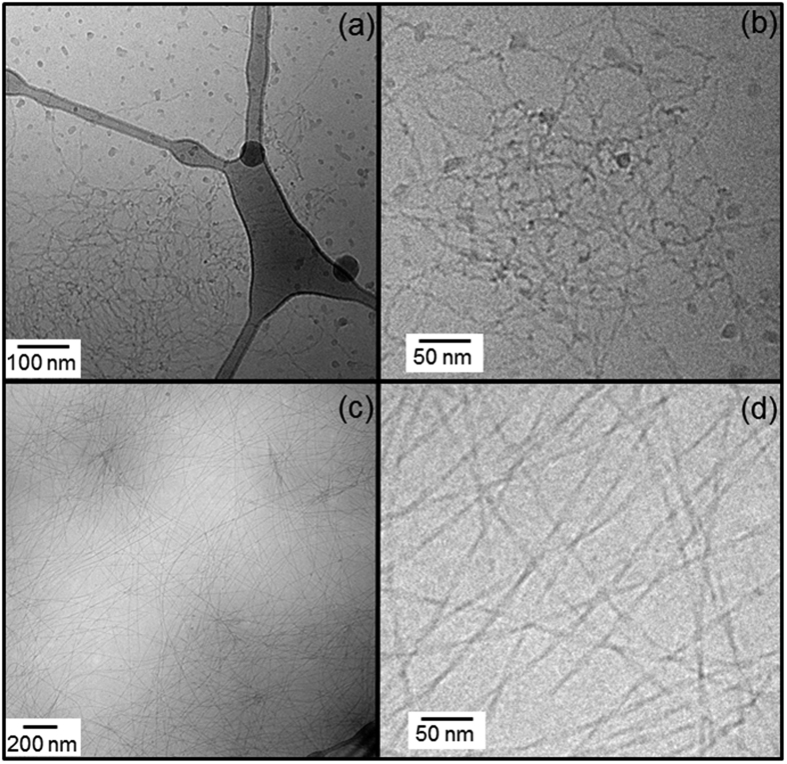
Cryo-TEM images for (**a,b**) 3 wt% peptide **1** and (**c,d**) 0.1 wt% peptide **2**.

Images from Cryo-TEM are shown in Fig. 7. These show the presence of fibrils for both samples, under conditions of imaging that was only possible above the *cac*. peptide **1** self-assembles in twisted tapes ~(6.4 ± 1.9) nm wide at 3 wt% peptide (Fig. 7a,b), while peptide **2** forms long twisted fibres ~(7.7 ± 1.4) nm wide at 0.1 wt% peptide concentration (Fig.7c,d).

**Figure 8:**
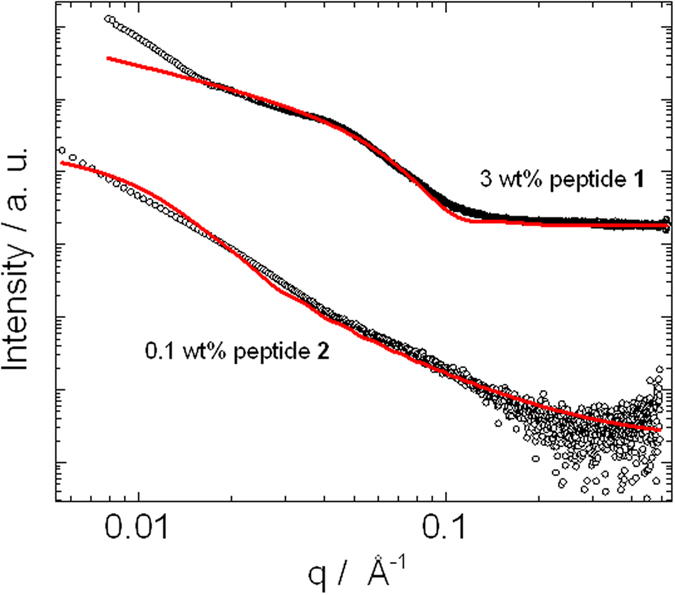
SAXS data measured for 3 wt% peptide **1** and 0.1 wt% peptide 2 fitted according to infinite cylinder form factor models. SAXS data for peptide **2** has been normalized by an arbitrary constant in order to enable the visualization of the data.

SAXS data for both samples is shown in Fig. 8. Consistent with the cryo-TEM images, SAXS data were fitted to form factors of fibrils (modelled using a Porod approximation for long cylinders^52^). Data for peptides **1** and **2** could be fitted with a cylinder radius of (3.0 ± 0.5) nm and (8.9 ± 5) nm respectively, which is consistent with the radius of the fibrils observed in the cryo-TEM images.

Preparation of samples as thin films for X-ray scattering provides a method to obtain diffraction patterns from small sample volumes, and with alignment imposed by the constraint of the planar nature of the substrate (with the possibility for additional in-plane alignment from the coating method). Figures S3 and S4 display the GISAXS and GIWAXS results measured for thin films of peptide **1** and peptide **2** prepared on silicon wafers. The spacings measured from this data are listed in Table 1, showing that there is an overlap of reflections between the GISAXS and the GIWAXS scattering ranges.

GISAXS data (Fig. S3 and Table 1) shows reflections intrinsic to the peptide film formation, since they are not observed in the SAXS curves in Fig. 8. Similarly, peaks in the GIWAXS intensity profile are slightly different from those measured in the XRD patterns (Fig. 6), probably due to the distinct nanostructure of the peptide film formed on the silicon wafer, to be discussed shortly.

The 2D GIWAXS pattern for peptide **1** in Figure S4 shows a preferential orientation. Reflexions at 41.2 and 18.1 Å are oriented perpendicularly to reflections at 5.4 and 4.4 Å, while the reflection at 11.3 Å is not oriented. The former reflections are oriented along the meridian, indicating lamellar ordering parallel to the substrate. The 2D spectrum for peptide **2** in Figure S4 is isotropic.

According to the indexation in Table 1, films of sample **1** on silicon wafers have a lamellar structure. The cell parameter is close to the length of one extended molecule, such that the β-strands are oriented perpendicularly to the lamellae.

According to the indexation in Table 1, the films of sample **2** do not present a particular order. Spacings at 87.5 and 14.9 Å do not provide enough information to describe a lamellar order. However, the GIWAXS data shows that molecules adopt a β-sheet secondary structure.

The effect of addition of peptides **1** or **2** on the secondary structure of Aβ(1–42) was investigated using CD spectroscopy and the kinetics were monitored. Figure S5 shows that the characteristic minimum associated with β-sheet structure (at 216 nm) in the CD spectra of Aβ(1–42) is significantly reduced upon addition of **1** or **2** and the reduction is larger for compound **2**. Figure S6 shows the time-dependence of the molar ellipticity of the β-sheet minimum. No significant change is observed for Aβ(1–42) or the two mixtures with **1** and **2** over the time scale studied (from 3 min to 50 hrs), i.e. no seeding effect was observed.

### Cell toxicity

**Figure 9:**
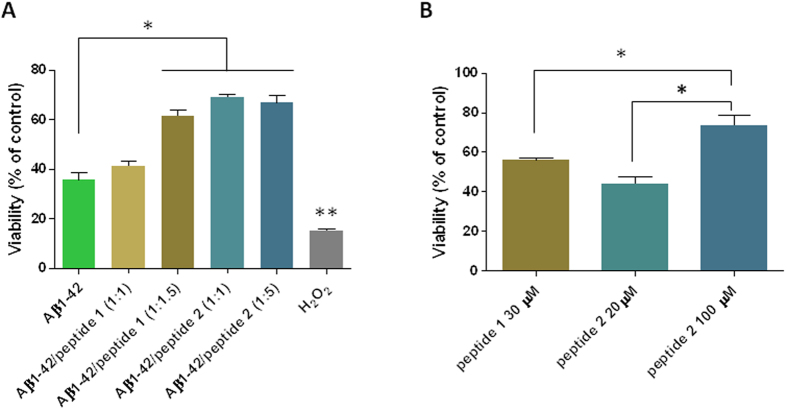
Peptides **1** and **2** reduced the toxic effect of Aβ(1–42) upon primary neurons. Panel A; neurotoxicity of Aβ(1–42) alone or co-incubated with peptide **1** or peptide **2** at the indicated molar ratios (Aβ: peptides). Peptide **1** at 1:1.5 and peptide **2** at 1:1 and 1:5 induced a significant reduction of Aβ toxicity. Bars represent the mean values ± SEM (standard error of the mean) of three MTS assays. *p < 0.01. H_2_O_2_ toxicity was significantly higher as compared to all other conditions (**p < 0.001). Data were analyzed by one way ANOVA followed by Bonferroni’s post-hoc test. Panel B; cell toxicity induced by peptides **1** and **2** alone at the concentrations used in co-incubations that reduced Aβ(1–42) toxicity. The means ± SEM of three assays are depicted. *p < 0.05 (one-way ANOVA and Bonferroni’s post hoc test).

The neurotoxicity of Aβ(1–42) and its mixtures with peptides **1** and **2** was assessed first using primary rat cortical neurons after 14–16 days of maturation. These cells were chosen since they are very sensitive to the effect of Aβ(1–42) oligomers^53^. Aβ(1–42) was incubated under conditions that yielded samples which were highly populated with oligomers instead of fibers, as described elsewhere^54^. When added to mature neurons at 20 μM and incubated for 20 h, Aβ(1–42) induced a remarkable loss in cell viability (~35% compared with vehicle) as measured with a MTS assay (Fig. 9a). Next, Aβ(1–42) at 100 μM was co-incubated for 72 h with peptides **1** and **2** during the oligomer formation process. Cell toxicity experiments were performed using peptide concentrations in a range below the *cac*. Peptide **1** had no effect at equimolarity but it improved viability by ~70% (p < 0.05) at a molar ratio of 1:1.5. Peptide **2** consistently reduced the toxicity of Aβ(1–42). At both molar ratios of co-incubation (1:1 and 1:5) there was nearly a ~2-fold significant increase in viability as compared to Aβ(1–42) alone. The recovery of viability by co-incubation with peptides **1** and **2** did not reach control values likely due to some extent of neurotoxicity of these peptides in the absence of Aβ(1–42). Notably, peptide **2** alone at the highest molarity (100 μM) showed a very mild toxic effect (~80% of control) (Fig. 9b).

Further cell toxicity experiments were performed using SH-SY5Y cells with 5 μM Aβ(1–42), 2.5 μM pure peptide and peptide to Aβ(1–42) ratios of 1:2, 1:5, 1:20 (peptide concentrations again below the *cac*s). Results obtained from MTS cell cytotoxicity assays are displayed in Figure S7. Addition of pre-aggregated Aβ(1–42) has a negative effect on cell viability as measured by the MTS assay, lowering the fluorescence intensity by approximately 30% when compared to untreated cells. Both **1** and **2** block the cytotoxic effect of Aβ(1–42) effect at the sub-stoichiometric concentration of 1:2 (peptide to Aβ(1–42) ratio). The anti-aggregation properties of the peptide CH_3_CONH_2_-QKLVFF-CONH_2_^18^ and free C-terminal KLVFF derivatives^19^ have been reported. Peptide **1** may have a similar protective effect at a 1:2 ratio. Lower concentrations of peptide **1** showed no protective effect, possibly due to high affinity binding of the compound to Aβ making it unavailable to act as an inhibitor by multiple interactions. Peptide **2** performed better than peptide **1** even at low molar ratio with respect to Aβ(1–42), with a suggestion of the multivalency effect reported for some other KVLFF-based inhibitors^55^,^56^. Cell viability remained above that of cells treated with Aβ at these ratios with only mild cytotoxicity even at high molarity.

## Discussion

We will first compare the influence of the C-terminus amidation (sample **1**), followed by the influence of the K-residue protection (sample **2**) on peptide self-assembly. In this process, results for sample **1** will be first compared to those previously published by us for the peptide NH_2_-KLVFF-COOH with uncapped termini^30^.

The fibres formed by NH_2_-KLVFF-COOH^30^ are ~10 nm wide, slightly thicker than fibres self-assembled from peptide **1**. The C-terminal amidated peptide fibres are twisted and shorter than those self-assembled from NH_2_-KLVFF-COOH. In addition, they do not form a macroscopic hard gel at 3 wt% peptide, as observed for NH_2_-KLVFF-COOH^30^.

Here we show that terminal capping limits longitudinal fibrillar growth and induces twisting of the fibres. This is in direct analogy with the results previously found for the C-terminal amidation of other amyloidogenic peptides^57^, where terminal capping affected fibril morphology in a similar way.

Boc protection of the K residue in the C-terminal amidated peptide induces a dramatic decrease in the *cac* by two orders of magnitude. The uncapped K residue influences self-assembly through electrostatic repulsion between neighbouring peptide molecules. Capping the K residue screens repulsive electrostatic interactions and promotes self-assembly. The features of fibres of sample **2** (net charge +1) resemble those observed for NH_2_-KLVFF-COOH fibres (net charge +1). It is likely that the effects on the fibril morphology induced by the amidation of the C-teminus are balanced by the protection of the K residue^30^. Grazing incidence X-ray scattering experiments indicate that **1** forms a planar lamellar structure in thin films, although the superstructure of **2** is less defined, both **1** and **2** have peaks associated with β-sheet structures in the film geometry.

In contrast to peptide **1**, peptide **2** exhibits a two-step aggregation process with hydrophobic collapse (in initially formed oligomers) occurring at a lower concentration than amyloid fibril formation. The oligomeric species in **2** above the two *cacs* are characterized by ESI MS and found to extend up to 20-mers and these are unstable in HFIP. Below its *cac*, compound **1** forms hardly any highly aggregated species, although these are stable in HFIP.

Both compounds **1** and **2** reduce the β-sheet content of Aβ(1–42) due to binding interactions. Both peptides are also able to restore the viability of both primary rat cortical neurons and SH-SY5Y neuroblastoma cells at peptide concentrations below the *cac* (i.e. in the absence of β-sheet fibrils) although they are less effective at lower peptide concentrations. These results are consistent with the anti-aggregation properties of KLVFF derivatives already reported^17^,^18^,^19^. Peptide **2** has a positive effect at low concentration, (1:1 molar ratio with 20 μM Aβ(1–42) in the assays with rat neurons) improving cell viability above that of cells treated with Aβ(1–42). These features are likely to be associated with the observed reduction in β-sheet content of Aβ(1–42) in mixtures with **2**. Thus, Boc capping of the lysine residue in a novel KLVFF derivative provides a valuable approach to reduce Aβ(1–42) β-sheet formation and neuronal toxicity.

## Methods

### Peptides

Peptide NH_2_-KLVFF-CONH_2_ was custom synthesized by Peptide Synthetics (UK) and was received as the TFA salt variant. Purity was >95% by HPLC in water/acetonitrile. Electrospray ionization (ESI) mass spectrometry (MS) results provided by the peptide supplier indicated a monoisotopic molar mass of 651.4 g mol^−1^ (average molar mass of 651.8 g mol^−1^, expected).

Peptide NH_2_-K(Boc)LVFF-CONH_2_ was custom synthesized by Peptide Synthetics (UK) and was received as the TFA salt variant. Purity was >95% by HPLC in water/acetonitrile. ESI MS results provided by the peptide supplier indicated a monoisotopic molar mass of 751.5 g mol^−1^ (average molar mass of 751.95 g mol^−1^, expected).

For CD experiments, Aβ(1–42) (H-Asp-Ala-Glu-Phe-Arg-His-Asp-Ser-Gly-Tyr-Glu-Val-His-His-Gln-Lys-Leu-Val-Phe-Phe-Ala-Glu-Asp-Val-Gly-Ser-Asn-Lys-Gly-Ala-Ile-Ile-Gly-Leu-Met-Val-Gly-Gly-Val-Val-Ile-Ala-OH) was purchased from American Peptide Inc. (Sunnyvale, USA). Purity was 95.14% based on RP-HPLC chromatography, M_w_ 4514.19 by ESI MS as provided by the supplier.

To prevent any pre-aggregation, Aβ(1–42) was first dissolved in hexafluoroisopropanol (HFIP) at a final concentration of 1 mg ml^−1 ^58,59,60. HFIP was then evaporated under a slow stream of N_2_. Aβ(1–42) was re-suspended in 20 mM Tris HCl 50 mM NaCl (pH 7.4) Tris buffer to attain a concentration 25 mM Aβ(1–42). When necessary, Aβ(1–42) was re-suspended in Tris buffer with sample **1** or sample **2** at molar ratios 1:1.2 and 1:1.6 for sample **1** and sample **2** respectively (i.e., 25 mM Aβ(1–42): 29 mM sample **1** or 25 mM Aβ(1–42): 39 mM sample **2**).

### Fluorescence Assays

8-anilo-1-naphthalenesulfonic acid (ANS) and thioflavin T (ThT) fluorescence were used to locate the critical aggregation concentration (*cac*). Spectra were recorded with a Varian Cary Eclipse fluorescence spectrometer with samples in 4 mm inner width quartz cuvettes. The ANS fluorophore is a probe sensitive to the hydrophobicity of its surrounding environment^34^, making it suitable to determine the *cac*. ANS assays were performed measuring spectra from 400 to 670 nm (λ_ex_ = 356 nm), using a 74 μM ANS solution to solubilize the peptide. ThT fluorescence depends on the formation of amyloid-like structures^36^,^37^ (β-sheet fibrils) and is used for amyloid fibril-forming peptides. For the ThT assay, the spectra were recorded from 460 to 600 nm using an excitation wavelength of λ_ex_ = 440 nm, and the peptide was dissolved in a 5.0 × 10^−3^ wt % ThT solution.

### Electrospray Ionization Mass Spectrometry

Samples were diluted to 0.029 wt % peptide in both H_2_O and HFIP:H_2_O (1:9; v/v). ESI mass spectra were acquired in positive ion mode on a Synapt G2-Si instrument (Waters Corporation, Wilmslow, UK). The employed Z-spray ESI source (Waters Corporation, Wilmslow, UK) was operated at 1.8 kV and the ESI sample solutions were infused at 10 μL/min using a KD Scientific syringe pump (KD Scientific Inc., Holliston, USA). The desolvation and source temperature were 150 °C and 80 °C, respectively, and the cone voltage was set to 40 V. ESI mass spectra were acquired in triplicate in sensitivity mode with the exception of higher resolution mass spectra, which were acquired once in resolution mode with ion mobility separation enabled. For the latter, wave velocity and wave height were set to 650 m/s and 40 V, respectively, and the helium and nitrogen flow rates were 180 and 90 mL/min, respectively. The acquired data was converted to mzML format using the msconvert tool from the ProteoWizard software package^61^. In-house developed software based on the pymzML software library^62^ was used to extract the integrated isotopomer signal intensities of interest from the converted data files. The integrated ion signal intensities of the monomer (*S*_*M*_) and all oligomers (*S*_*O*_) were summed for each acquired spectrum to determine the total ion signal intensity of the analysed peptide species (*S*_*T*_):


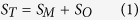


The relative extent of oligomerisation (*E*_*O*_) was determined by dividing the integrated ion signal intensities of the oligomers (*S*_*O*_) by the total ion signal intensity of the analysed peptide (*S*_*T*_):


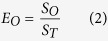


### Circular Dichroism (CD) Spectroscopy

CD spectra were recorded using a Chirascan spectropolarimeter (Applied Photophysics, UK). Peptide solutions were placed in a cover slip cuvette (1 × 10^−2^ or 0.1 mm thick), spectra presented being with absorbance A < 2 at any measured point with a 0.5 nm step, 1 nm bandwidth, and 1 s collection time per step. The CD signal from the background was subtracted from the CD data of the sample solutions. The circular dichroism of the samples was measured as a function of the time for the sample containing pure 26 μM Aβ(1–42) or mixtures of Aβ(1–42) with sample **1** or sample **2**. CD experiments were performed using a Quartz cuvette with 1mm path length.

### Fourier Transform Infra-Red (FTIR) Spectroscopy

Spectra were recorded using a Nexus-FTIR spectrometer equipped with a DTGS detector. Samples were measured using an ATR configuration, with a PEARL liquid cell, or a transmission configuration, with the sample placed between two CaF_2_ plate windows (spacer 1.2 × 10^−2^ mm thick). Spectra were scanned 128 times over the range of 900–4000 cm^−1^.

### X-ray Diffraction (XRD)

Measurements were performed on stalks prepared by drying filaments of solutions of sample **1** and sample **2**. The stalk for sample **1** was prepared from a 3 wt% solution but for the solutions containing sample **2**, a 1 wt% solution was used. Solutions of the mixtures were suspended between the ends of wax-coated capillaries and dried. The stalks were mounted onto the four axis goniometer of an Oxford Diffraction Gemini Ultra instrument. The sample-detector distance was 44 mm. The X-ray wavelength was λ = 1.54 Å. The wavenumber scale (*q* = 4πsinθ/λ, where 2θ is the scattering angle) was geometrically calculated. The detector was a Sapphire CCD.

### Cryogenic-Transmission Electron Microscopy (Cryo-TEM)

Imaging was carried out using a field emission cryo-electron microscope (JEOL JEM-3200FSC), operating at 200 kV. Images were taken in bright field mode using zero loss energy filtering (omega type) with a slit width of 20 eV. Micrographs were recorded using a Gatan Ultrascan 4000 CCD camera. The specimen temperature was maintained at −187 °C during the imaging. Vitrified specimens were prepared using an automated FEI Vitrobot device using Quantifoil 3.5/1 holey carbon copper grids with a hole size of 3.5 μm. Just prior to use, grids were plasma cleaned using a Gatan Solarus 9500 plasma cleaner and then transferred into an environmental chamber of a FEI Vitrobot at room temperature and 100% humidity. Thereafter 3 ml of sample solution was applied on the grid and it was blotted twice for 5 seconds and then vitrified in a 1/1 mixture of liquid ethane and propane at temperature of −180 °C. The grids with vitrified sample solution were maintained at liquid nitrogen temperature and then cryo-transferred to the microscope.

### Small-Angle and X-Ray Scattering (SAXS)

Synchrotron SAXS experiments were performed on beamline BM29 at the ESRF (France), using a BioSAXS robot, and on beamline BM26B (DUBBLE, ESRF).

On BM29 solutions were loaded into the 96 well plate of an EMBL BioSAXS robot, and then injected via an automated sample exchanger into a quartz capillary (1.8 mm internal diameter) in the X-ray beam. The quartz capillary was enclosed in a vacuum chamber, in order to avoid parasitic scattering. After the sample was injected into the capillary and reached the X-ray beam, the flow was stopped during the SAXS data acquisition. BM29 operated with an X-ray wavelength λ = 1.03 Å (12 keV). The images were captured using a PILATUS 1 M detector, while data processing was performed using dedicated beamline software ISPYB (BM29).

On BM26B, the sample was sandwiched between two mica windows with a 1 mm Teflon space. The sample-to-SAXS detector distance was 3.16 m using a wavelength of 1.033 Å. A Dectris-Pilatus 1 M detector with a resolution of 981 × 1043 pixels and a pixel size of 172 × 172 μm was used to acquire the 2D SAXS scattering patterns. Standard corrections for sample absorption and background subtraction were performed. The data were normalized to the intensity of the incident beam (in order to correct for primary beam intensity fluctuations) and were corrected for absorption and background scattering. Diffraction from silver behenate were used to calibrate the wavevector scale of the scattering curve.

### Grazing incidence small angle X-ray scattering (GISAXS) and grazing incidence wide angle X-ray scattering (GIWAXS)

GISAXS and GIWAXS experiments were performed on beamline I07 of the Diamond Light Source (UK). Amounts 20 μl of peptide solution using a 2:1 methanol:chloroform mixture (1 wt% sample **1** or 2.5 × 10^−3^ wt% sample **2**), were first spread on silicon wafer substrated by manually rocking the substrate, and immediately spin coated for 1 minute. This process was repeated 5 times per sample. The nanostructure of the films was characterized using GISAXS and GIWAXS experiments. Films on silicon wafers were positioned inside a custom built experimental chamber. A Pilatus 2 M detector was used to collect the scattered X-rays. GISAXS was collected for grazing incident angles (4.3 × 10^−4^–0.6211) Å^−1^, while GIWAXS was collected for grazing incident angles (3.9 × 10^−3^–5.2529) Å^−1^.

### Primary neuronal cultures

Protocols for the handling of rats used for neuronal cultures followed the Guide for the care and use of Laboratory Animals, National Research Council (US) and were reviewed and approved by the Committee for the Care and Use of Laboratory Animals of Fundacion Instituto Leloir. Neuronal cultures were obtained from E16 rat embryos as described^63^ with modifications. Briefly, brain cortical tissue was dissected free of meninges and suspended in 0.5 ml of Ca^2+^ and Mg^2+^ –free Hank’s balanced solution (Gibco). Tissue was then incubated with 4 ml of 0.25% trypsin in a 37 °C water bath for 20 min with agitation. After digestion, tissue was centrifuged at 1000 rpm for 2 min, suspended in DMEM (Gibco) containing 10% fetal calf serum (FCS) (Internegocios) and incubated at 37 °C for 10 min. The pellet was washed with DMEM 10% FCS and allowed to rest for 10 min. Medium was replaced with 1 ml Neurobasal medium (Gibco) and triturated with a 10 ml plastic pipette. After incubation for 10 min, the suspension was centrifuged at 1000 rpm for 1 min and suspended in Neurobasal medium supplemented with B27 (Gibco), 0.5 mM glutamine, 50 IU/ml penicillin and 50 mg/ml streptomycin (Gibco) (NB medium). The cellular suspension was plated in poly-L-lysine coated 96-well plates (4 × 10^4^ cells/well) and incubated under 5% CO_2_ at 37 °C. Seventy-two hours after plating, in order to inhibit astrocyte proliferation, 50% of culture medium was removed and the cells were treated with 1 μM Ara-C for 3 days. After this period, fresh NB medium was added to the neurons. Such treatment yielded neuronal purity of 90–95%. Cultures were maintained for 14–16 days to allow neuronal differentiation.

### Neurotoxicity Assays with Primary Neurons

For cell toxicity experiments, peptides stock solutions were made in HFIP at 600 μM for Aβ(1–42) and 2.5 mM for peptides **1** and **2**, respectively. Aliquots were dried under a gentle stream of N_2_ and re-suspended in 20 mM Tris-HCl, pH 7.4, containing 50 mM NaCl (working buffer). Aβ(1–42) was incubated at 100 μM alone or co-incubated with peptide **1** and peptide **2**. Molar ratios were 1:1 and 1:1.5 for Aβ(1–42): peptides **1** and **2**. After 72 h in a humidified chamber at room temperature, peptide suspensions were added to cultured neurons in 100 μl of neurobasal/B27 medium to a final Aβ(1–42) concentration of 20 μM and incubated for 20 h at 37 °C. Cell viability was measured using a (3-(4, 5-dimethylthiazol-2-yl)-5-(3-carboxymethoxyphenyl)-2-(4-sulfophenyl)-2H-tetrazolium) (MTS) reduction assay. Twenty μl of MTS solution containing phenazine ethyl sulfate were added to wells, incubated for 1 h and absorbance was measured at 490 nm. Viability was expressed as the O.D. relative to neurons treated with working buffer alone. As a positive control for neuronal damage, cells were exposed to 0.05% H_2_O_2_. Data of three MTS assays were analyzed by one-way ANOVA and Bonferroni post hoc test with Graph Pad Prism v. 4.0. All results represent the mean ± SEM and p < 0.05 was considered statistically significant.

### Neurotoxicity Assays with SH-SY5Y Cells

SH-SY5Y neuroblastoma cells were maintained in a 1:1 ratio of Ham’s F12 and Dulbecco’s Modified Eagle Medium with 10% foetal calf serum and penicillin/streptomycin at 37 °C and 5% CO_2_. At 85–95% confluence the cells were detached from their growth substrate using trypsin and the growth medium was removed following centrifugation of the cells at 500x*g* for five minutes. The cells were re-suspended in a serum-free medium (Optimem with penicillin/streptomycin) and distributed at 20,000 cells per well in a 96-well plate then incubated at 37 °C, 5% CO_2_ for a further 24 hours. The medium was replaced with Optimem, which contained 5 μM of Aβ(1–42) (Ultrapure, HFIP treated, from rPeptide) pre-aggregated for 24 hours at room temperature in sterile PBS. Aβ(1–42) was not present in control wells. Solutions containing sample **1** or sample **2** were also added to the wells at this time at 1:2, 1:5 and 1:20 ratios of peptide to Aβ(1–42) (2.5, 1 and 0.25 μM), plus a set of wells at 2.5 μM inhibitor in the absence of Aβ(1–42). Cells were incubated for a further 24 hours as above and then assayed for cell survival using an MTS based assay (CellTiter 96 AQueous One Solution Cell Proliferation Assay from Promega).

## Additional Information

**How to cite this article**: Castelletto, V. *et al*. Self-Assembly and Anti-Amyloid Cytotoxicity Activity of Amyloid Beta Peptide Derivatives. *Sci. Rep.* **7**, 43637; doi: 10.1038/srep43637 (2017).

**Publisher's note:** Springer Nature remains neutral with regard to jurisdictional claims in published maps and institutional affiliations.

## Supplementary Material

Supplementary Information

## Figures and Tables

**Figure 1:**
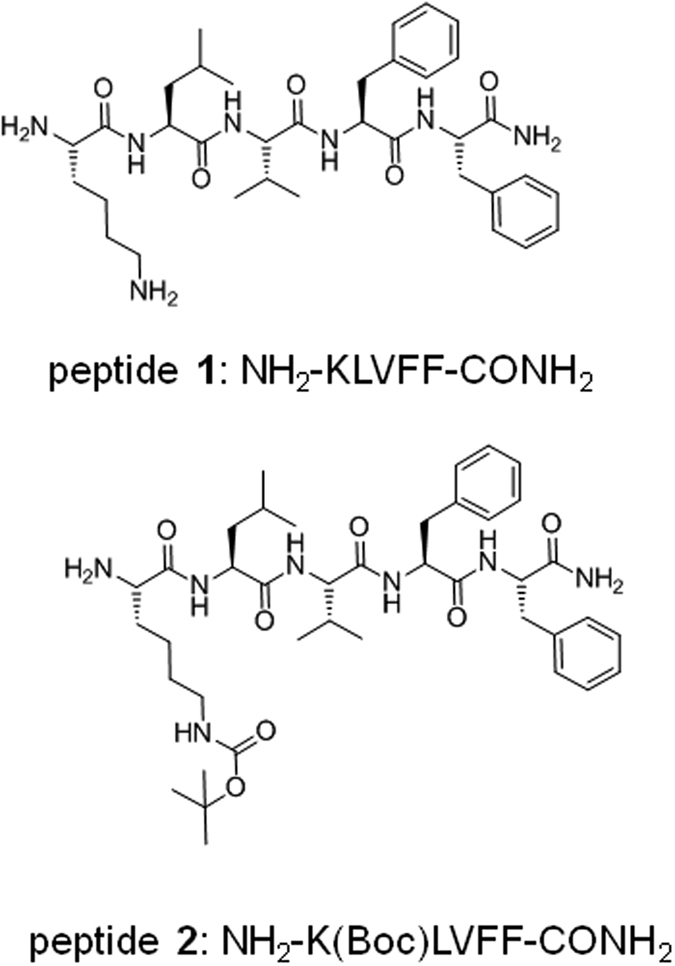
Molecular structures of peptide **1** and peptide **2**.

**Table 1:** GISAXS and GIWAXS spacings measured from data in Figures S3 and S4.

Sample	GISAXS spacings/	GIWAXS spacings/Å	Origin
1 wt% peptide **1**	62.2		Long spacing–third order
	41.2	41.2	Long spacing–second order
	18.1	18.1	First order (mol. length ~17 Å)
	11.3	11.3	β-sheet stacking distance
		4.8	β-strand spacing
		4.4	β-strand spacing splitting
		1.9	Intramolecular spacing
		1.6	“
		1.5	“
2.3 × 10^−3^ wt% peptide **2**	87.5		
	14.9	14.9	
		9.2	β-sheet stacking distance
		4.7	β-strand spacing
		1.9	Intramolecular spacing
		1.4	“
