# Supplementary material for: Self-Assembly and Anti-Amyloid Cytotoxicity Activity of Amyloid beta Peptide Derivatives
Source: Sci Rep. 2017 Mar 8;7:43637. doi: 10.1038/srep43637 (PMC5341572; doi:10.1038/srep43637)
Supplement: Supplementary Information [file srep43637-s1.doc]

**Supplementary Information**

**Self-Assembly and Anti-Amyloid Cytotoxicity Activity of Amyloid beta Peptide Derivatives**

V. Castelletto,*^a^* P. Ryumin,*^a^* R. Cramer,*^a^* I.W. Hamley*,*^a^* M. Taylor,*^b^* D. Allsop,*^b^* M. Reza,*^c^* J. Ruokolainen,*^c^* T. Arnold,*^d^* D. Hermida-Merino,*^e^* C. I. Garcia,*^f^* M. C. Leal*^f^* and E. Castaño*^f^*

*^a^School of Chemistry, Pharmacy and Food Biosciences. University of Reading. Whiteknights, Reading RG6 6AD, United Kingdom.*

*^b^Division of Biomedical and Life Sciences, Faculty of Health and Medicine*

*Lancaster University, Lancaster LA1 4YQ, United Kingdom.*

*^c^Department of Applied Physics, Aalto University School of Science, Aalto FI-00076, Finland.*

*^d^Diamond Light Source Ltd., Harwell Science and Innovation Campus, Didcot OX11 0DE, United Kingdom.*

*^e^European Synchrotron Radiation Facility, ESRF, 71 avenue des Martyrs, 38000 Grenoble, France.*

*^f^ Fundación Instituto Leloir and Instituto de Investigaciones Bioquímicas de Buenos Aires, Consejo Nacional de Investigaciones Científicas y Técnicas, Buenos Aires, Argentina*


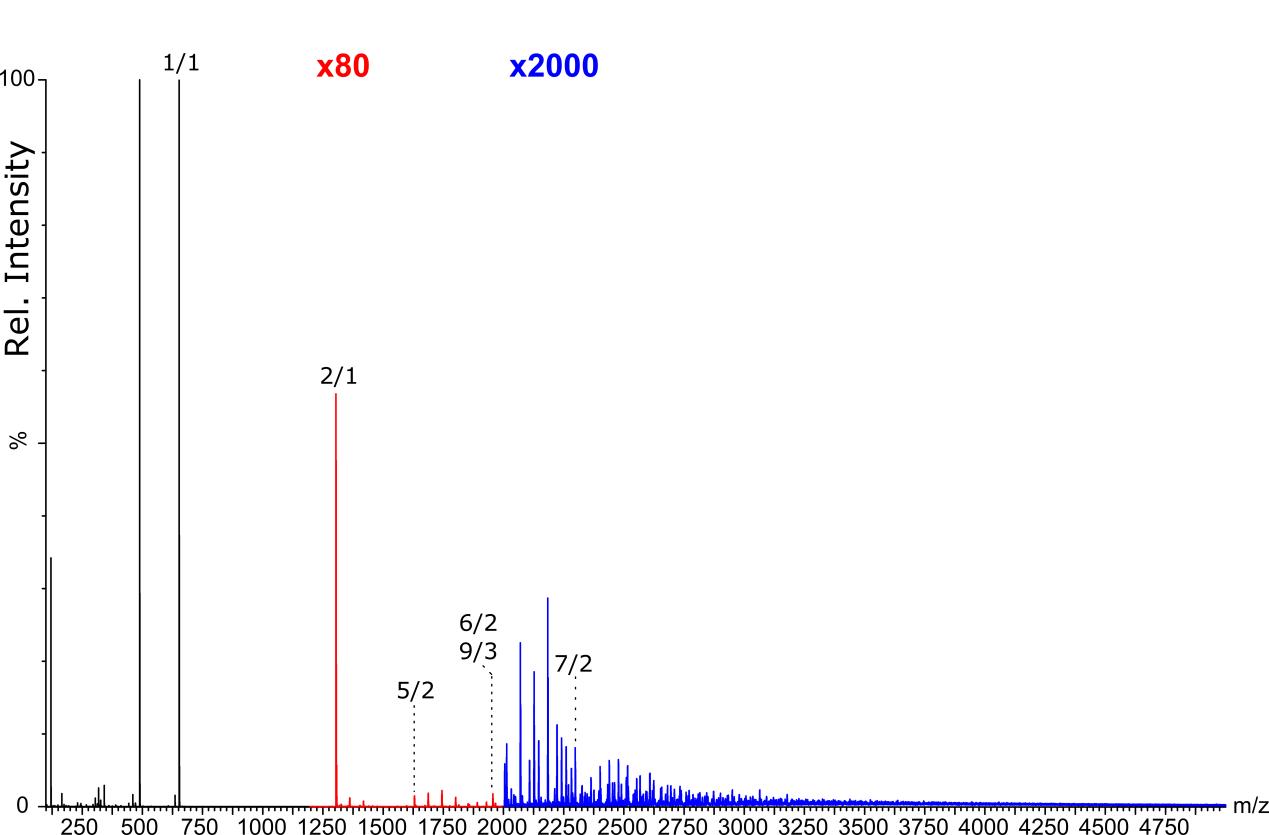


**Figure S1.** ESI mass spectrum showing peptide aggregation for peptide **1**. The peaks are annotated by the number of monomers n over the charge z (n/z). The spectrum sections shown in red and blue are magnified 80 and 2000 times, respectively.


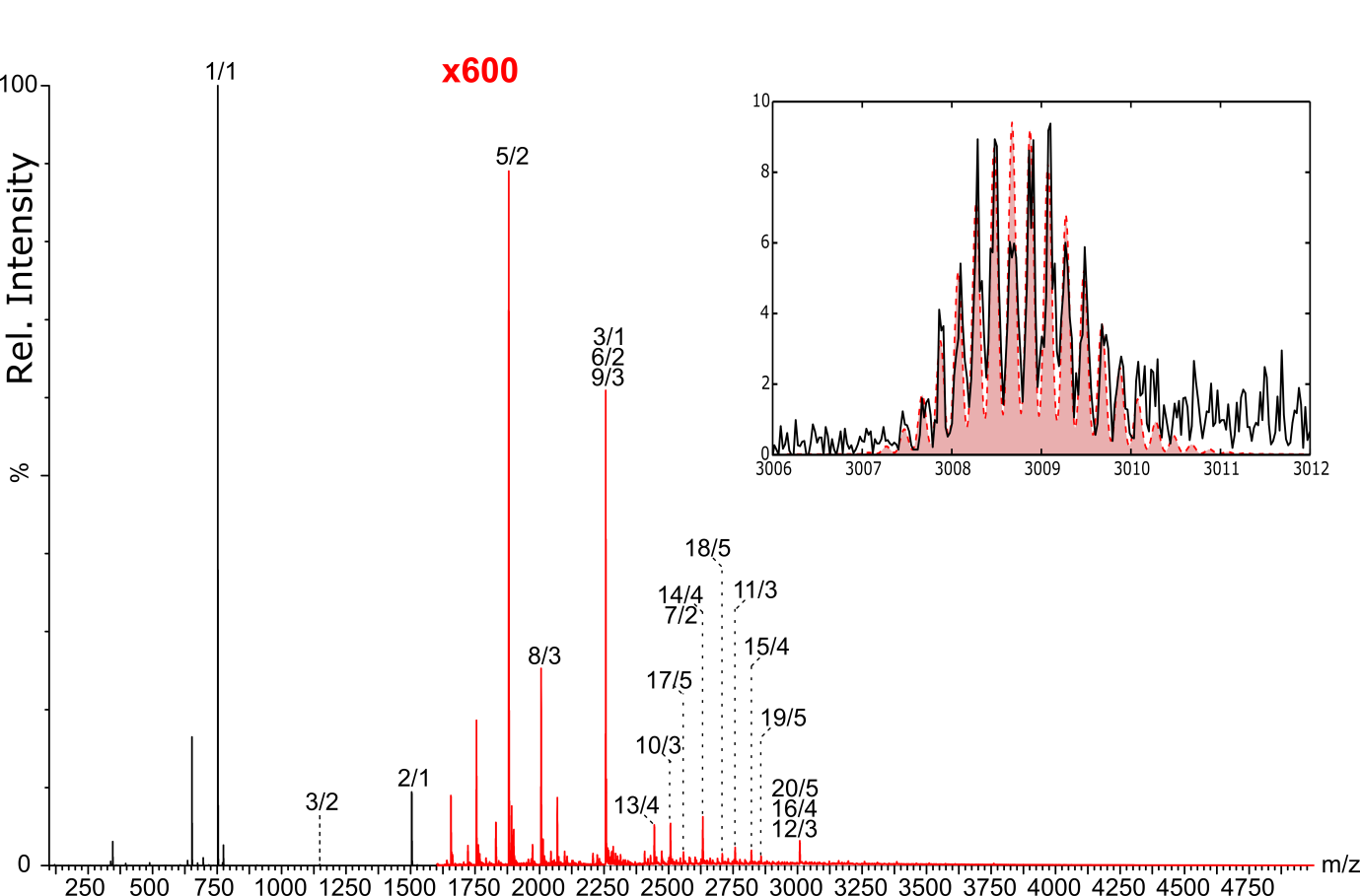


**Figure S2.** ESI mass spectrum showing peptide aggregation for peptide **2**. The peaks are annotated by the number of monomers n over the charge z (n/z). The spectrum section shown in red is magnified 600 times. All labelled oligomers were unambiguously characterized by employing ion mobility separation and MS data acquisition at higher resolution. The inset shows the MS ion signal of the 20/5 oligomer (solid black) filtered out from the overlapping ion signals of oligomers with different charge states (z values) by ion mobility separation. The theoretical isotopomer distribution of this oligomer (dashed red with the area under the curve in pink) is also depicted.

**Figure S3.** GISAXS data measured for silicon wafers coated with thin films made from solutions of (a) 1 wt% peptide **1** or (b) 2.5×10^-3^ wt% peptide **2** in 2:1 methanol: chloroform.

**Figure S4.** GIWAXS data measured for silicon wafers coated with thin films made from solutions of (a) 1 wt% peptide **1** or (b) 2.5×10^-3^ wt% peptide **2** in 2:1 Methanol: Chloroform. The insets display the 2D GIWAXS spectra. Samples are the same as those used in Fig. S3.

**Figure S5**. CD spectra for solutions containing 25 μM Aβ(1-42) or Aβ(1-42) mixtures with peptide **1** or **2** at molar ratios 1:1.2 and 1:1.6 respectively (25 μM Aβ(1-42): 29 μM peptide **1** and 25 μM Aβ(1-42): 39μM peptide **2**). The time *t* is indicated after sample solubilisation (t=0 min).

**Figure S6**. Molar ellipticity at 215 nm obtained from the CD data, as a function of time, for the samples under the conditions indicated in the figure. The time at which the sample was solubilised is t = 0.


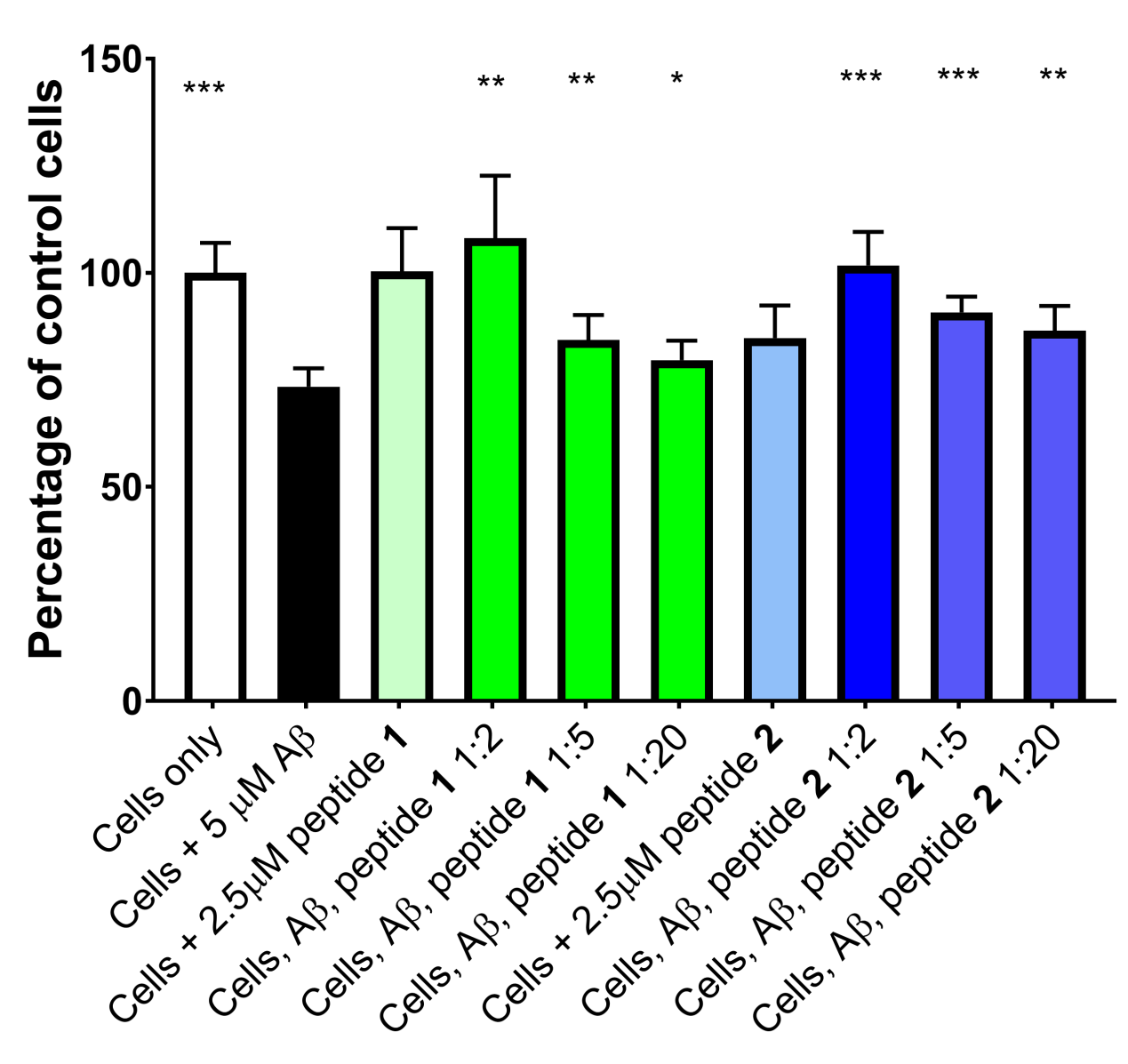


**Figure S7.** Cell assay results for SH-SH5Y cells grown in the presence of Aβ(1-42) show a loss in viability (dark green column) compared to control cells (white column). Addition of peptide **1** at a ratio of 1:2 (compound to Aβ) returns cell viability to 100% of control, whereas a 1:5 and 1:20 ratio are not very effective (light green bars). Peptide **2** also rescues the cells at a 1:2 ratio and has a positive effect at 1:5 and 1:20. However, unlike peptide **1** (grey bar), there is a negative effect of adding peptide **2** to cells alone (light blue bar). These assays are performed using peptide concentrations below *cac*. Analysis from t-test: * = p<0.05, ** = p<0.01 and *** = p<0.001.
